# Effectiveness of check-up 35 for detecting targeted diseases in Germany

**DOI:** 10.1186/s12875-026-03307-4

**Published:** 2026-04-10

**Authors:** Andreas Kommer, Julia Weinmann-Menke, Karel Kostev, Christian Labenz

**Affiliations:** 1 Department of Internal Medicine I, University Medical Center of the Johannes Gutenberg-University, Langenbeckstraße 1, Mainz, 55131 Germany; 2 Department of Nephrology, University Medical Center of Heidelberg, Heidelberg, Germany; 3 Epidemiology, IQVIA, Frankfurt, Germany

**Keywords:** Health check-up, Prevention, Primary care, Dyslipidemia, Arterial hypertension

## Abstract

**Background:**

The Check-up 35 has been a key element of primary prevention in German general practice since 1989. However, evidence on its effectiveness in detecting target diseases remains limited. Therefore, we analyzed the detection rates of the target diseases of the Check-up 35 in a large cohort of persons treated in German primary care.

**Methods:**

In a retrospective cohort study using data from 1,216 German primary care practices (IQVIA™ Disease Analyzer, 2019–2023), we compared Check-up 35 participants with non-participants via 1:5 propensity score matching. We analyzed diagnoses of arterial hypertension, diabetes mellitus, dyslipidemia, and chronic kidney disease (CKD) within three months post-check-up versus a random visit in controls. Medication prescriptions following diagnosis served as a secondary outcome.

**Results:**

We compared 51,829 participants with 259,145 matched non-participants. Participation was associated with higher diagnosis rates: hypertension (HR 1.56), diabetes (HR 1.69), dyslipidemia (HR 3.24), and CKD (HR 2.07). The effect was especially pronounced for dyslipidemia in individuals under 40 (HR 5.10). Prescription rates after diagnosis were similar or lower in the Check-up group.

**Conclusion:**

Check-up 35 is effective in early detection of key chronic diseases. Broader participation should be encouraged to improve prevention.

## Background

According to the data from the European Observatory on Health Systems and Policies cardiovascular disease is the leading cause of death in Germany. A substantial number of these deaths are considered avoidable by prevention [1]. Large multinational studies have established hypertension, dyslipidemia, diabetes, body-mass index and current smoking as the main risk factors for cardiovascular disease and death [2]. More recently, chronic kidney disease (CKD) has been established as an independent risk factor for cardiovascular morbidity and mortality as well [3]. Although randomized controlled trials and consecutive meta-analysis have failed to provide evidence for an effect on mortality, health check-ups are an established part in a lot of western health care systems [4, 5].

In Germany, the screening examination “Check-up 35” was established in 1989 to identify major risk factors for morbidity and mortality in the population. In short, the examination includes a history and physical examination, a blood test for dyslipidemia and fasting glucose as well as a urine dipstick test for protein, glucose, nitrite, leucocytes and hemoglobin. All Germans under statutory health insurance over the age of 35 are eligible every 3 years and all between ages 18 and 34 are eligible once for this health check.

Most published studies regarding the Check-up 35 examination evaluate participation in certain socio-economic groups [6–9]. However, studies evaluating the effectiveness of the Check-up 35 are lacking. Studies in other healthcare systems, namely the United Kingdom and Denmark, have found mixed results regarding the effectiveness of health check-ups in the detection of targeted risk factors [10–12]. To evaluate the effect of participation in the Check-up 35 examination on the detection rates of targeted diseases and its impact on drug prescriptions, we conducted a retrospective cohort study comparing participants and non-participants in German general practitioner offices.

## Methods

### Database

This retrospective cohort study was based on data from the Disease Analyzer database (IQVIA), which contains drug prescriptions, diagnoses, and basic medical and demographic data obtained directly and in anonymous format from computer systems used in the practices of general practitioners and specialists. The database covers approximately > 1200 office-based primary care physicians in Germany. It has previously been shown that the panel of practices included in the Disease Analyzer database is representative of general and specialized practices in Germany [13].

### Study population

This study included adult individuals (≥ 18 years) with at least one visit to one of 1,216 primary care physicians in Germany between January 2019 and December 2023. We chose this time span given that there was a change in the “Einheitlicher Bewertungsmaßstab” (EBM) *(German for “standardized payment for health services”)* for Check-up 35 starting in January 2019. For subsequent analyses, we only included individuals who underwent a Check-up 35 in this time span (*n* = 1,365,445) and matched this group to patients who did not undergo a Check-up 35 (*n* = 4,841,157). In this study, a complete Check-up 35 was defined by a code of all of the following EBM numbers: 01732 for Check-up 35 history and physical examination, 32,880 for urine test, 32,881 for fasting glucose and 32,882 for lipid panel. These examinations primarily screen for hypertension, diabetes, dyslipidemia and kidney disease. Hypertension screening relies on non-standardized in-office blood pressure measurements, which have known limitations including anxiety-related elevation, inconsistent patient preparation, and lack of longitudinal data. Abnormal findings are typically followed by home blood pressure monitoring using a diary. Diabetes screening is usually limited to fasting glucose testing, which is less sensitive than oral glucose tolerance testing and is not diagnostic on its own; abnormal results generally prompt HbA1c measurement. Dyslipidemia assessment includes fasting total cholesterol, LDL, HDL, and triglycerides, which represent the gold standard for lipid evaluation. Screening for chronic kidney disease is restricted to a non-standardized urine dipstick test for protein, glucose, hemoglobin, leukocytes, and nitrite, which has poor sensitivity and detects only substantial proteinuria.

To make the groups comparable and well matched, we excluded individuals who underwent additional urinary and blood tests and those with an observation time of less than 12 months prior to the index date. This was important to further exclude all individuals with a prior coded diagnosis of diabetes mellitus (ICD-10: E10-E14), dyslipidemia (ICD-10: E78), hypertension (ICD-10: I10) or chronic kidney disease (CKD) (ICD-10: N18). We also excluded individuals with ischemic heart disease (ICD-10: I25) or stroke (ICD-10: I60-I69), because in these individuals undercoding of the aforementioned diagnoses of interest seems likely. Afterwards, we conducted a propensity score matching (1:5) between individuals with and without a Check-up 35 focusing on age, sex and a coded diagnosis of obesity (coded before Check-up 35).

### Study outcomes

The primary study outcomes were the initial diagnoses of type 2 diabetes mellitus (ICD-10: E11) arterial hypertension (ICD-10: I10), dyslipidemia (ICD-10: E78) or CKD (ICD-10: N18) within three months of the respective index date. Due to quarterly billing in German practices, we assume that diagnoses made during a Check-up 35 are coded within three months at the latest, as they are relevant for billing purposes. The index date was defined as the time point of the Check-up 35 in the Check-up group and a randomly selected visit date between 2019 and 2023 in the group without a Check-up.

As a secondary outcome, we investigated the prescription rates of medication for the aforementioned diagnoses within six months of the date of the initial diagnoses. For this purpose, we noted the following medication: For type 2 diabetes mellitus: metformin (Code A10BA02), Insulin (Code A10A), SGLT2 Inhibitors (A10BK), DPP4 inhibitors (A10BH); For arterial hypertension: ACE inhibitors (Code C09A, C09B), Angiotensin receptor blockers (Code C09C, C09D), Diuretics (Code C03), Betablockers (Code C07), Calcium channel blockers (Code C08); for dyslipidemia: statins (Code C10AA); for CKD: ACE inhibitors (Code C09A, C09B), Angiotensin receptor blockers (Code C09C, C09D) and SGLT2 Inhibitors (A10BK).

### Ethics

The “Disease Analyzer” database used as a source of data in this study contains anonymized electronic patient records. As patient data was analyzed in aggregated form without any individual data being available, ethical approval and informed consent was waived by the ethics committee of the Landesärztekammer Rheinland-Pfalz and also and in the past also by the ethics committee of the medical faculty of the Christian-Albrechts-University of Kiel within another project dealing with the exactly same database (AZ 413/21). All methods were carried out in accordance with the Declaration of Helsinki and its later amendments.

### Statistical analyses

The quality of the propensity score matching was evaluated via standard mean differences (SMD). A SMD < 0.1 was considered adequate balance. The three-month cumulative incidence of diabetes mellitus, hypertension, dyslipidemia, and CKD were studied using the Kaplan-Meier method. This method was needed due to adjust for the difference in the follow-up time between the two cohorts. Finally, an univariable Cox regression analysis was conducted to assess the association between Check-up 35 and each diagnosis. These models were conducted separately for women and men and by age. Results of the Cox regression model are displayed as hazard ratios (HRs) and 95% confidence intervals (CIs). The respective number needed screen to find one case of the diagnosis of interest within the general population was calculated by dividing 100 by the difference in the cumulative incidences between both groups at 3 months. Due to multiple comparisons (12 models), p-value of < 0.001 was considered statistically significant. Analyses were conducted using SAS version 9.4 (SAS Institute, Cary, USA).

## Results

### Basic characteristics of the study sample

**Fig. 1:**
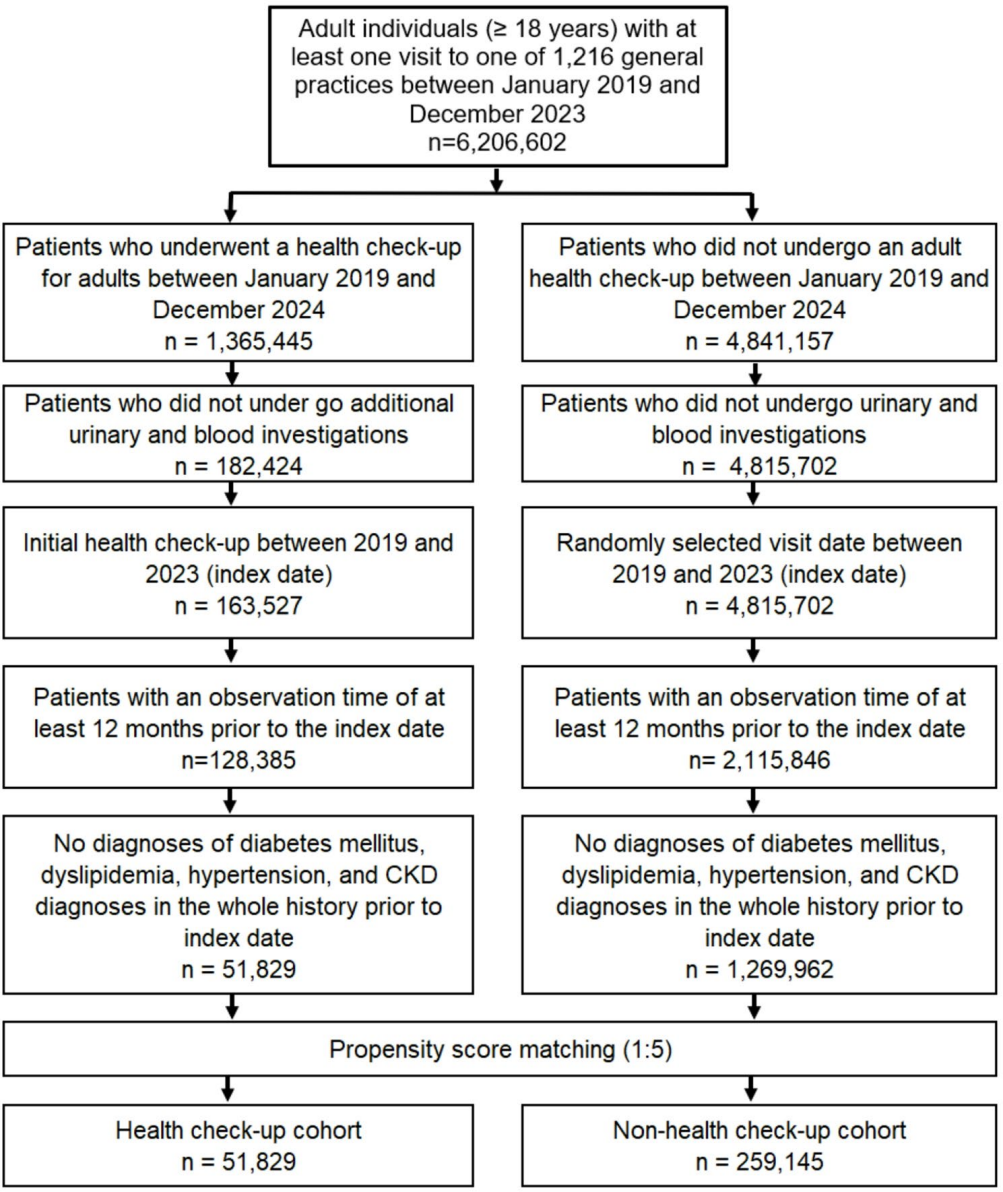
Selection of study patients

In this study 51,829 individuals with a Check-up 35 were matched with 259,145 individuals without a Check-up 35 (Fig. 1). The individuals were well matched as displayed in Table 1. The cohorts had a mean age of 47.8 (SD 13.7) years and all age groups had sufficient case numbers. Nearly 60% of the cohorts were female and check-up examinations were evenly distributed among the individuals over the years. Obesity was only coded in 5.7% of the cohorts. All characteristics are displayed in Table 1.

**Table 1:** Baseline characteristics of the study sample (after 1:5 propensity score matching)

Variable	Proportion among patients with check-up 35 (*N*, %) *N* = 51,829	Proportion among patients without check-up 35 (*N*, %) *N* = 259,145	SMD
Age (Mean, SD)	47.8 (13.7)	47.8 (13.7)	0.000
Age *≤* 40 years	16,817 (32.5)	84,075 (32.4)	-0.011
Age 41–50 years	13,565 (26.2)	67,812 (26.2)	
Age 51–60 years	12,610 (24.3)	63,000 (24.3)	
Age > 60 years	8,837 (17.0)	44,258 (17.1)	
Female	31,036 (59.9)	155,221 (59.9)	0.000
Male	20.793 (40.1)	103,924 (40.1)	
Year of study inclusion			
2019	13,296 (25.7)	66,442 (25.7)	-0.001
2020	8.928 (17.2)	44,687 (17.2)	
2021	11,849 (22.9)	59,162 (22.8)	
2022	9,026 (17.4)	45,131 (17.4)	
2023	8,730 (16.8)	43,723 (16.9)	
Obesity diagnosis	2,966 (5.7)	14,736 (5.7)	-0.001

Proportions of patients in N, % given, unless otherwise indicated. *SD* Standard deviation, *SMD* Standardized mean difference

### Incidence of subsequent diagnoses after a check-up examination

**Fig. 2:**
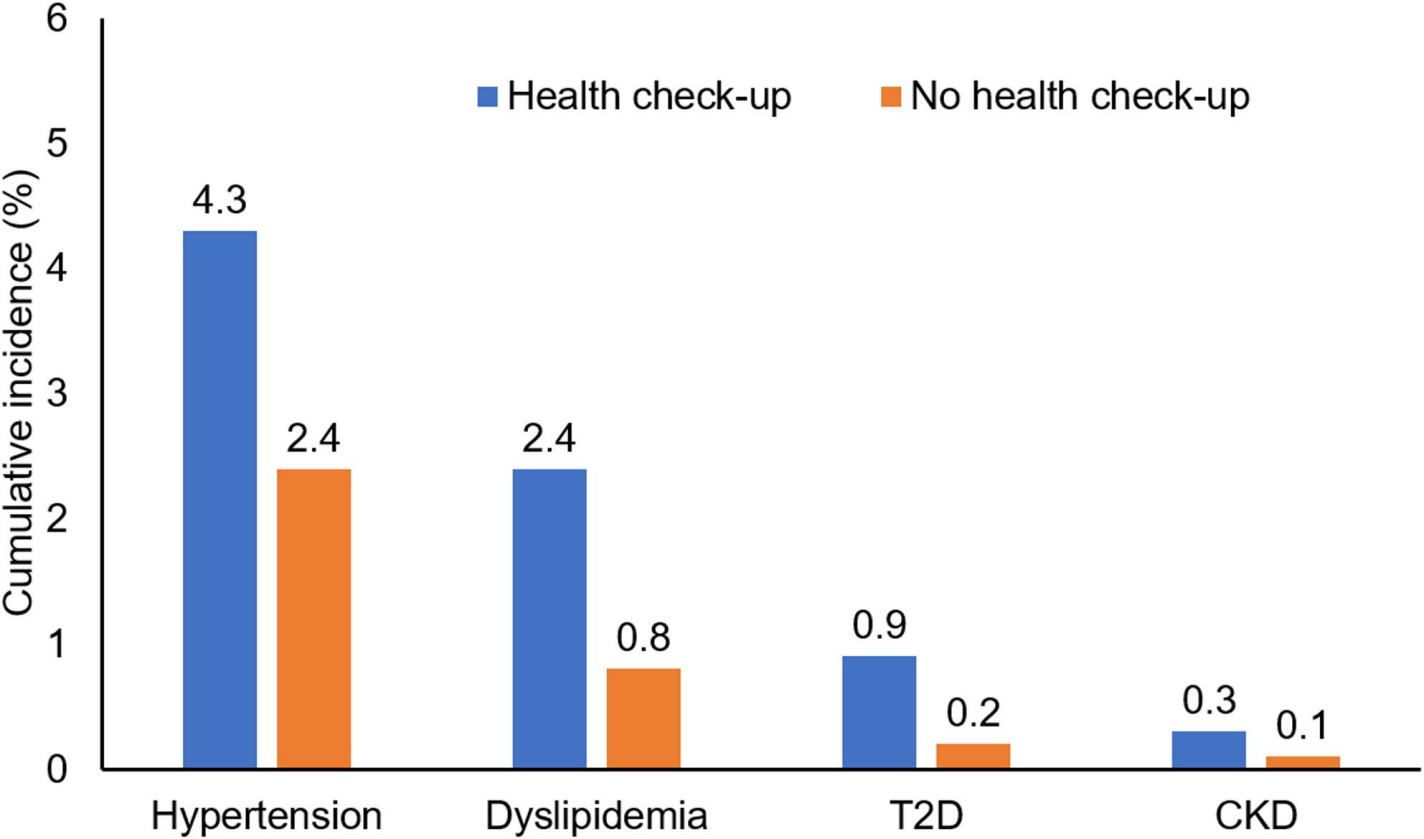
Cumulative 3-month-incidence of diabetes mellitus, hypertension, dyslipidemia, and chronic kidney disease in patients with and without health check-up

Three months after a check-up examination (Check-up 35 cohort) or the index date (controls), there was a significantly higher rate of new coded diagnoses of arterial hypertension, dyslipidemia, type 2 diabetes mellitus and CKD in patients who participated in the Check-up 35 compared to controls (Fig. 2). The respective numbers needed to screen to detect one case of the targeted disease in the general population were as follows: 53 (arterial hypertension), 63 (dyslipidemia), 143 (type 2 diabetes mellitus), 500 (CKD). This association remained significant in univariable Cox regression analyses across various age groups as well as female and male individuals. Notably, especially for the diagnoses of dyslipidemia and CKD, participation in a Check-up 35 examination was strongly associated with a subsequent diagnosis. The association between Check-up 35 participation and a dyslipidemia diagnosis was strongest in individuals below or equal to the age of 40 years with a more than 5-fold increased risk (Table 2).

**Table 2:** Association between health check-up and subsequent diagnoses of diabetes mellitus, dyslipidemia, hypertension, and CKD in patients followed in general practices in Germany (univariable Cox regression models)

Patient subgroup	Incidence (cases per 1000 patient years) among individuals with health check-up	Incidence (cases per 1000 patient years) among individuals without health check-up	HR (95% CI)
Type 2 diabetes mellitus			
Total	10.08	6.26	1.69 (1.58–1.81)*
Age *≤* 40 years	4.43	2.34	1.84 (1.52–2.22)*
Age 41–50 years	8.88	4.91	1.85 (1.61–2.13)*
Age 51–60 years	11.38	7.70	1.58 (1.40–1.79)*
Age > 60 years	19.35	14.29	1.47 (1.31–1.65)*
Female	8.67	5.58	1.60 (1.47–1.75)*
Male	12.31	7.29	1.82 (1.65–2.01)*
Dyslipidemia			
Total	48.54	11.85	3.24 (3.13–3.36)*
Age *≤* 40 years	34.53	4.08	5.10 (4.68–5.56)*
Age 41–50 years	45.40	9.52	3.48 (3.33-3,74)*
Age 51–60 years	58.13	23.23	2.77 (2.60–2.95)*
Age > 60 years	63.02	31.35	2.45 (2.27–2.63)*
Female	43.70	15.26	3.10 (2.95–3.25)*
Male	56.23	9.24	3.45 (3.27–3.64)*
Hypertension			
Total	42.65	27.89	1.56 (1.51–1.61)*
Age *≤* 40 years	20.52	11.78	1.79 (1.64–1.95)*
Age 41–50 years	38.48	23.87	1.62 (1.51–1.73)*
Age 51–60 years	51.36	36.83	1.44 (1.35–1.52)*
Age > 60 years	75.64	54.69	1.40 (1.32–1.49)*
Female	38.68	26.08	1.49 (1.42–1.55)*
Male	48.96	30.61	1.66 (1.58–1.75)*
CKD			
Total	4.14	1.99	2.07 (1.85–2.31)*
Age *≤* 40 years	1.30	0.61	2.28 (1.60–3.26)*
Age 41–50 years	2.15	1.07	1.87 (1.40–2.50)*
Age 51–60 years	4.30	1.78	2.37 (1.89–2.96)*
Age > 60 years	11.45	6.64	1.73 (1.48–2.02)*
Female	3.69	1.87	1.97 (1.70–2.28)*
Male	4.83	2.16	2.20 (1.87–2.60)*

*p < 0.001

### Prescription of medication following a diagnosis of diabetes, dyslipidemia, arterial hypertension or CKD

To examine the frequency of medication prescriptions following one of the aforementioned diagnoses, we analyzed the six-month period after the initial diagnosis. In patients with a coded diagnosis of dyslipidemia, 15.5% in the Check-up 35 cohort received a prescription compared to 27.4% in the cohort without a Check-up 35. The prescription rates regarding diabetes or arterial hypertension medication were comparable between individuals with and without a Check-up 35 (Diabetes: Check-up 46.7% vs. no Check-up 43.8%; arterial hypertension: Check-up 79.4% vs. no Check-up 73.9%). After a diagnosis of CKD, only 13.1% and 10.7% of the patients from the Check-up and no Check-up group received a prescription of medication, respectively. A granular presentation of the respective prescribed drugs is presented in Table 3.

**Table 3:** Percentage of prescribed medications after diagnosis

	Check-up 35 cohort	No Check-up 35 cohort
Dyslipidemia		
Therapy at all Statins Other	15.5% 14.8% 0.7%	27.4% 26.4% 1.0%
Type 2 diabetes mellitus		
Therapy at all Metformin Insulin SGLT2 inhibitors DDP4 inhibitors Other	46.7% 37.0% 2.2% 3.2% 3.0% 1.3%	43.8% 28.9% 3.3% 3.3% 4.4% 3.9%
Arterial hypertension		
Therapy at all Diuretics Beta-blockers Calcium channel blockers ACE inhibitors Angiotensin receptor blockers	79.4% 2.3% 12.5% 6.5% 36.7% 21.4%	73.9% 5.8% 18.2% 7.7% 24.7% 17.5%
Chronic kidney disease		
Therapy at all ACE inhibitors Angiotensin receptor blockers SGLT2 inhibitors	13.1% 6.2% 6.9% 0.0%	10.7% 4.9% 5.8% 0.0%

## Discussion

Check-up 35 was introduced into the German healthcare system in 1989 and updated in 2019. Our current study demonstrates that participation in Check-up 35 is associated with a higher subsequent coding rate of related diagnoses such as type 2 diabetes mellitus, arterial hypertension, dyslipidemia, and CKD. Notably, the initiation rate of pharmacological treatment for these conditions was comparable or even lower than that observed in matched control individuals during the first six months following the respective diagnosis.

Health check-ups are well established, particularly in Western countries. In Germany, there is a specific focus on metabolic diseases and the early detection of cancer. However, for the majority of check-up examinations, dedicated studies — particularly randomized controlled trials — are lacking to evaluate the effectiveness of these programs for early detection of the respective conditions. Our current study adds to the literature by demonstrating, using a robust design, that participation in the updated 2019 version of Check-up 35 is associated with a higher likelihood of being diagnosed with the aforementioned conditions. The likelihood was strongest for the detection of dyslipidemia and CKD, which highlights the occult nature of these diseases. Considering that approximately 56.6% of men and 60.5% of women in Germany suffer from dyslipidemia and about 11.9% of the adult population as a whole suffer from CKD [14–16]. These findings are mostly in line with findings in similar studies from NHS in the United Kingdom showing that participation in health checks substantially increase the detection of dyslipidemia and only slightly increases the detection of hypertension and diabetes [11, 17]. This could show that there is already high awareness of hypertension and diabetes and low awareness for dyslipidemia and CKD in primary care.

Although we excluded all individuals with pre-existing coded diagnoses prior to their participation in Check-up 35, and even excluded those with conditions such as ischemic heart disease due to their high likelihood of having hypertension or diabetes, our findings must be interpreted in context of the study design. We cannot entirely rule out the possibility that individuals with a higher a priori risk of, for example, arterial hypertension were more likely to be offered Check-up 35 by their physician, introducing a potential selection bias. Additionally, we cannot exclude that individuals who participated in Check-up 35 may have had greater health awareness or were of higher socio-economic status compared to the control group. However, a recently published study evaluating the integration of a liver fibrosis score into Check-up 35 found that nearly 50% of participants did not attend the recommended follow-up appointment with a gastroenterologist, suggesting that disease awareness in this population may not be significantly higher than in the general population [18].

One important finding of our study was that the initiation rate of pharmacological treatment for these conditions was comparable to or even lower than that observed in matched control individuals, showing at least some therapeutic inertia when it comes to asymptomatic randomly diagnosed disease. We can only hypothesize on reasons for this finding. The most likely explanation might be that individuals participating in Check-up 35 were in earlier disease stages so that a lifestyle intervention without medication was chosen as a first step. Additionally, diseases might only be coded in the control group when there is intention to initiate pharmacological treatment. This might be especially true for dyslipidemia. Yet, considering the indication for treatment of dyslipidemia with lipid lowering therapy depends on the overall risk for cardiovascular disease. We hypothesize that, as has previously been shown, at-risk patients with dyslipidemia are often undertreated in Germany [19].

Another noteworthy finding was that only about 10% of patients with an initial diagnosis of CKD received a prescription of an ACE inhibitor or Angiotensin receptor blocker and none received a prescription of an SGLT2 inhibitor. Although we are unable to provide more granular information on the disease stage of CKD, it seems that CKD is massively undertreated in German primary care. This is a worrisome finding given that CKD is treatable and end-stage kidney disease is associated with increased cardiovascular disease, a poor prognosis, has detrimental effects on health-related quality of life and is a huge economic burden for the health-care system [20, 21]. Therefore, measures should be intensified in sharing the recently published evidence on the positive studies investigating SGLT2-inhibotors on the progression of CKD [22, 23]. This is especially troublesome as CKD diagnosed during Check-up 35 is likely diagnosed due to the detection of proteinuria on urine dipstick. Urine dipstick testing is often only positive in patients with macroproteinuria (i.e. greater than 300 mg a day), which is a major risk factor for the progression of kidney disease and for cardiovascular risk in general [24]. The overall low detection rate of CKD in our cohort highlights the limitations of urine dip stick testing to screen for kidney disease given the high prevalence of CKD of around 10% within the population [25–27]. These findings underscore the need for more comprehensive screening methods, potentially incorporating blood-based parameters such as serum creatinine to calculate estimated glomerular filtration rate (eGFR) and better assess kidney disease.

As to our knowledge, this is the first study evaluating the effectiveness of Check-up 35 in detecting targeted diseases. The biggest strength of our study is the large sample size allowing thorough matching and leading to robust analyses. However, there are also limitations that have to be acknowledged. Our analysis relies on ICD-10 codes making the study vulnerable for undercoding. However, this bias may be balanced across both groups. When interpreting the findings of our study, one has to consider that the patients in the control group were visiting their primary care physician for some reason, which is not coded in Disease Analyzer. This might lead to sicker patients in the no check-up group than in the check-up group, who might have attended to their primary care physician only for the Check-up 35 yet were otherwise healthy. Another limitation is that we are unable to investigate the impact of Check-up 35 on a hard clinical endpoint such as death. Unfortunately, the Disease Analyzer database does not cover death and the respective causes for death. Future studies are needed to delineate the usefulness of Check-up 35 in this context. Furthermore, the Disease Analyzer does not include other interactions with the German healthcare system in general, i.e. hospital or specialist treatment, so we cannot account for diagnosis and prescription made outside of the > 1200 office-based primary care practices. We chose a period of three months for a coded diagnosis and of six months for a drug prescription after the index date. This period might have missed some additional diagnoses that might have been coded after an extensive workup. However, a potential longer period might have introduced bias due to diagnoses due to different reasons and circumstances. Last, the Check-up 35 is a screening program embedded within the German healthcare system. Therefore, our findings cannot be directly generalized to other countries with different healthcare structures.

Our current study demonstrates that participation in Check-up 35 in Germany is associated with a higher subsequent coding rate of related diagnoses such as type 2 diabetes mellitus, arterial hypertension, dyslipidemia, and CKD. It also shows the high therapeutic inertia regarding the treatment of dyslipidemia and CKD regardless of participation in a screening program. Nevertheless, our findings indicate the usefulness of Check-up 35 and the general population should be encouraged to participate to prevent future complications.

## Data Availability

Data cannot be shared publicly due to ethical restrictions, but raw are available on reasonable request from K.K.

## Abbreviations

CI: Confidence interval
CKD: Chronic kidney disease
EBM: Einheitlicher Bewertungsmaßstab
HR: Hazard ratio
ICD: International Statistical Classification of Diseases and Related Health Problems
SD: Standard deviation
SMD: Standardized mean difference
